# Insights into HPLC-MS/MS Analysis, Antioxidant and Cytotoxic Activity of *Astragalus fruticosus* against Different Types of Cancer Cell Lines

**DOI:** 10.3390/ph15111406

**Published:** 2022-11-14

**Authors:** Mohamed Fayez Dekinash, Tarek M. Okda, Ehab Kotb Elmahallawy, Fathy Kandil El-Fiky, Gamal Abd El Hay Omran, Emil Svajdlenka, Naief Dahran, Manal F. El-Khadragy, Wafa A. Al-Megrin, El Moataz Bellah Ali El Naggar

**Affiliations:** 1 Department of Pharmacognosy, Faculty of Pharmacy, Damanhour University, Damanhour 22511, Egypt; 2 Department of Biochemistry, Faculty of Pharmacy, Damanhour University, Damanhour 22511, Egypt; 3 Department of Zoonoses, Faculty of Veterinary Medicine, Sohag University, Sohag 82524, Egypt; 4 Department of Pharmacognosy, Faculty of Pharmacy, Delta University for Science and Technology, Mansoura 35511, Egypt; 5 Department of Natural Drugs, Faculty of Pharmacy, Masaryk University, 60300 Brno, Czech Republic; 6 Department of Anatomy, Faculty of Medicine, University of Jeddah, Jeddah 21959, Saudi Arabia; 7 Department of biology, College of Science, Princess Nourah bint Abdulrahman University, P.O. Box 84428, Riyadh 11671, Saudi Arabia

**Keywords:** *Astragalus fruticosus*, HPLC–MS/MS profiling, colorectal cancer, cytotoxic activity

## Abstract

Plants from the genus *Astragalus* are gaining attention for their pharmacological importance. However, the information available regarding the HPLC–MS/MS chemical profile of *A. fruticosus* is inadequate. In this study, we performed HPLC–MS/MS analysis using electrospray ionization (ESI) and atmospheric pressure chemical ionization (APCI). We tentatively identified 11 compounds in the *A. fruticosus* methanolic extract, including five flavonoidal and six saponin glycosides. The extract showed moderate antioxidant activity with 21.05% reduction in DPPH UV absorption. The preliminary cytotoxic screening against seven human cancer cell lines using 100 μg/mL extract showed prominent cytotoxic potential against colorectal cancer HCT–116 with 3.368% cell viability. It also showed moderate cytotoxic potential against prostate (DU–145), ovarian (SKOV–3) and lung (A–549) cancer cell lines with cell viability of 14.25%, 16.02% and 27.24%, respectively. The IC_50_ of the total extract against HCT–116 and DU–145 cell lines were 7.81 μg/mL and 40.79 μg/mL, respectively. The observed cytotoxicity of the total methanolic extract from the leaves against colorectal cancer might facilitate future investigations on cytotoxic agent(s) for disease management.

## 1. Introduction

The genus *Astragalus* is the largest genus in the family *Leguminosae* (*Fabaceae*), with over 3000 species of annual and perennial small shrubs and herbs. It is one of the largest and most diverse genera among angiosperms [1]. *Astragalus* has been used as a medicinal plant for over 1000 years. Radix Astragali (*A. membranaceus* and *A. mongholicus*) is an ancient and well-known Chinese traditional herbal medicine that is used to enhance resistance against bacterial and viral infections, treat immunological disorders, stimulate the circulatory system, control excessive sweating, and as a diuretic, hepatoprotective and heart tonic [2]. The chemical composition of *Astragalus* uniformly consists of major active compounds, including polysaccharides, flavonoids (free or glycosidic forms) and saponins [3]. *Astragalus* contains both cycloartane and oleanane saponins with the latter being relatively rare [2]. The saponin-rich purified mixture isolated from *A. corniculatus* showed protective activity against the hamster myeloid graffi tumor [4]. The saponins extracted from *Astragalus hamosus* showed anticancer activity against breast carcinoma cell lines [2].

Using *Astragalus*-based Chinese medicine in combination with chemotherapy for treating colorectal and lung cancer increases the treatment efficacy, reduces the side effects and improves the patients’ quality of life [5]. *Astragalus* extracts also have other biological activities, including antioxidant [6], hepatoprotective [7] and immunomodulatory activities [8]. The saponins isolated from *A. hamosus*, *A. kahiricus* and *A. peregrinus* showed dose-related modulation of lymphocyte proliferation [9]. There are 37 species of *Astragalus* in Egypt as described by Tackholm [10], while Boulos recorded only 32 species [11]. To our knowledge, until early 2022, previous phytochemical and biological research on members of genus *Astragalus* in Egypt included the following 15 species: *A. sieberi* [12,13,14], *A. hamosus* [9], *A. kahiricus* [7,9,15], *A. vogelii* [16], *A. eremophilus* [16,17], *A. bombycinus* [18,19], *A. peregrinus* [8,19,20], *A. annularis* [21], *A. trimestris* [21], *A. tomentosus* [22,23,24], *A. spinosus* [25,26,27], *A. tribuloides* [28], *A. trigonus* [29,30,31], *A. alexandrinus* [29,32] and *A. cremophilos* [33]. A recent study discussed potential somatic embryogenesis and micropropagation of the endangered plant *Astragalus fruticosus* to ensure its conservation. The study also identified the presence of luteolin and kaempferol flavonoids, pyrogallol, protocatechuic acid, *p*–coumaric acid, chlorogenic acid, ferulic acid and ellagic acid by comparing the high-performance liquid chromatography-ultraviolet (HPLC–UV) chromatograms of the plant’s ethanolic extract and reference compounds. *Astragalus fruticosus* ethanolic extract showed α–glucosidase inhibitory activity (IC_50_ = 44.8 µg/mL) and exhibited cytotoxic activity against breast (MCF–7) and leukemia (HL–60) cell lines (IC_50_ of 28.3 µg/mL and 49 µg/mL, respectively) [34].

Notably, the combination of the HPLC chromatographic resolution and the structural information by MS and MS^2^ allows tentative identification of phytochemicals in plant extracts [35,36,37,38]. The information available regarding the HPLC–MS/MS chemical profiling of *A. fruticosus* and their activity on different cell lines is limited. Here we performed a preliminary cytotoxic screening of the *A. fruticosus* Forssk methanolic extract against a panel of seven cancer cell lines and determined its IC_50_ against the most sensitive cell lines. Additionally, we performed high performance liquid chromatography coupled to tandem mass spectrometry (HPLC–MS/MS) analysis of the methanolic extract, which is ideal for analyzing phytochemicals, including saponins and flavonoidal glycosides, due to its high sensitivity and selectivity.

## 2. Results and Discussion

### 2.1. HPLC–MS/MS Identification of Compounds

**Figure 1:**
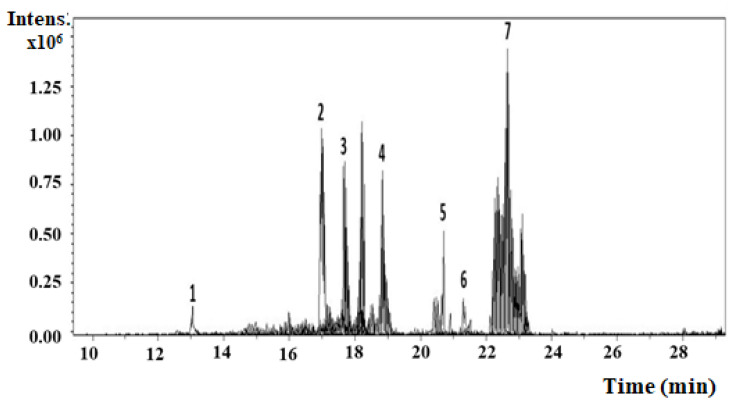
The extracted ions chromatogram of the identified compounds using ESI-Ve ionization mode in tandem mass spectrometry.

Using HPLC–MS/MS analysis, we tentatively identified 11 compounds from *Astragalus fruticosus*. Figure 1 shows the extracted ion chromatogram of the seven compounds identified using ESI-Ve ionization mode in the tandem mass spectrometry, and their HPLC/MS–MS data and spectra are listed in Table 1. The HPLC/MS–MS data and spectra of compounds identified using atmospheric pressure chemical ionization (APCI)+Ve ionization mode in the MS–MS are listed in Table 2. The mass spectra for tentatively identified compounds using HPLC–MS/MS showing glycon daughter peaks are provided in the Supplementary Figures S1–S11.

#### 2.1.1. Compounds Identified Using ESI Mode

**Figure 2:**
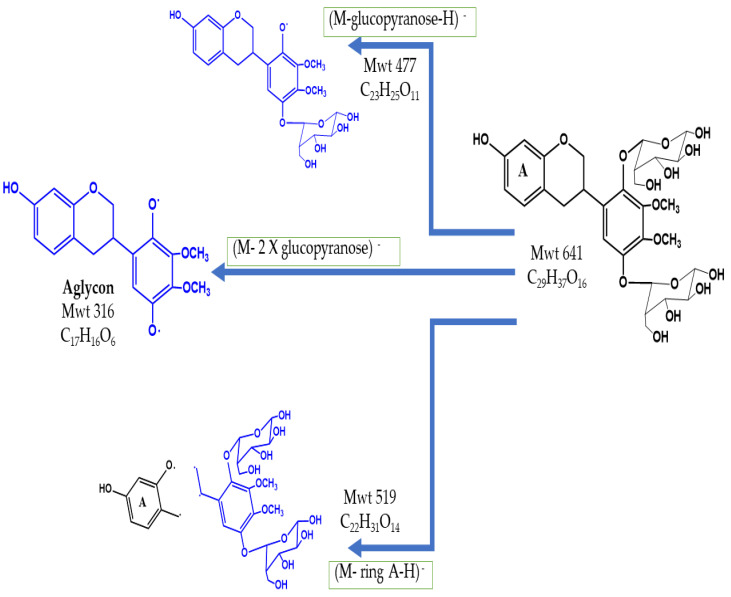
The structure and fragmentation of 5–hydroxy isomucronulatol–2′,5′–di–*O*–glucoside.

The peak at Rt 13 and *m*/*z* 640 was identified as the (M–H)^−^ of 5–hydroxy isomucronulatol–2′,5′–di–*O*–glucoside(C_29_H_37_O_16_). This was further confirmed by MS^2^, where the loss of one glucopyranosyl unit (M–162), two sugar units and ring A resulted in the base peak at *m*/*z* 477, glycon signal at *m*/*z* 316 and the daughter peak at *m*/*z* 520, respectively. The 5–hydroxy isomucronulatol–2′,5′–di–*O*–glucoside was previously identified as a flavonoid component in Radix Astragali [39], and its structure and fragments are illustrated in Figure 2. 

**Figure 3:**
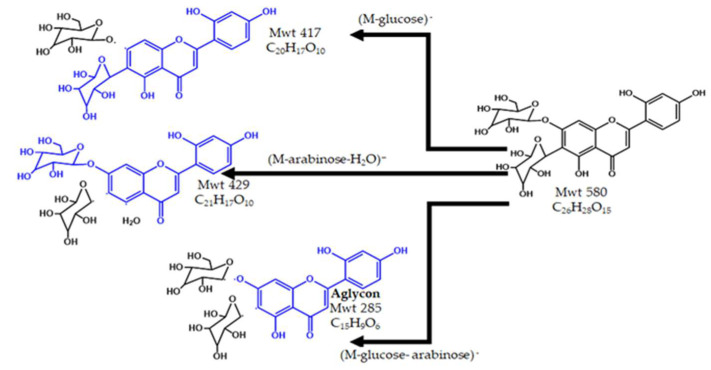
The structure and fragmentation of 2′,4′–trihydroxy–flavone–8–*C*–*α*–arabinopyranoside–7–*O*–*β*–glucopyranoside.

The peak at Rt 17.1 and *m*/*z* 579 was identified as the (M–H)^−^ of 2′,4′–trihydroxy–flavone–8–*C*–α–arabinopyranoside–7–*O*–β–glucopyranoside (C_26_H_27_O_15_)^−^ that was previously isolated and fully characterized from *A. bombycinus* [18]. The MS^2^ spectrum showed a distinctive base peak at *m*/*z* 284.8 representing the genin compartment due to the loss of the sugar units (M–(162) glucopyranosyl–(132) arabinopyranosyl)^−^. The daughter peaks at *m*/*z* 447 (M–132)^−^ and *m*/*z* 429 (M–132–18)^−^ were due to the loss of the arabinopyranosyl moiety. The loss of a glucose caused the daughter peak at *m*/*z* 417. Figure 3 shows the chemical structure and fragmentation of 2′,4′–trihydroxy–flavone–8–C–α–arabinopyranoside–7–*O*–β–glucopyranoside.

**Figure 4:**
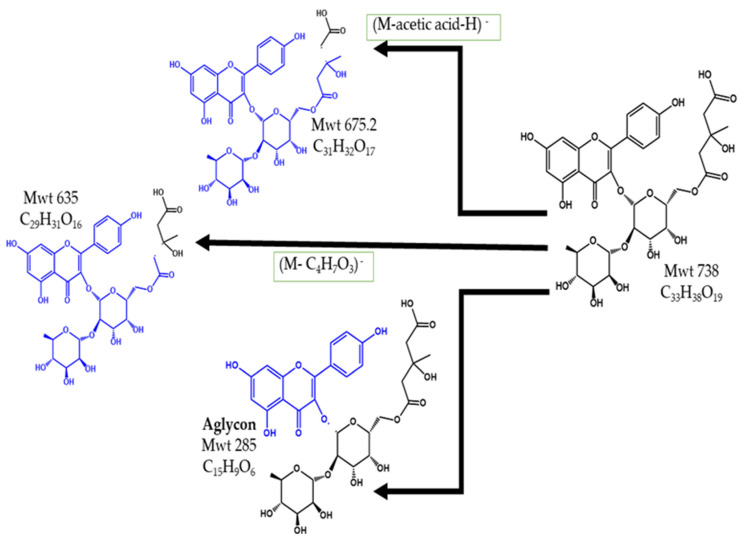
The structure and fragments of KaeHMG.

The compound eluted at Rt 17.7 and *m*/*z* 737.5 using ESI -Ve was identified as the (M–H)^–^ of kaempferol–3–*O*–α–L– rhamnopyranosyl–(1→2)–[6–*O*–(3–hydroxy–3–methyl– glutaryl)–β–d–galactopyranoside] (KaeHMG), a flavonoidal glycoside that was previously identified in *A. depressus* [41], *A. monospessulanus subsp. Illyricus* [41] and *A. gombiformis* [40]. The MS^2^ spectrum demonstrated a peak at *m*/*z* 593 representing the loss of the terminal rhamnopyranose unit, which was previously documented in the MS^2^ spectrum for KaeHMG when the phenolic components of *A. gombiformis* were analyzed [40]. The peak at *m*/*z* 635 was due to the cleavage of the 3–hydroxy–3–methyl glutaryl moiety through McLafferty rearrangement at the carboxylic group attached to the sugar moiety. A peak at *m*/*z* 675 is probably due to loss of acetic acid from the 3–hydroxy–3–methyl glutaryl moiety and the formation of a stable tertiary carbocation attached to the methyl and hydroxyl groups. The aglycon kaempferol is observed at *m*/*z* 285. Figure 4 demonstrates the chemical structure and fragmentation of KaeHMG.

**Figure 5:**
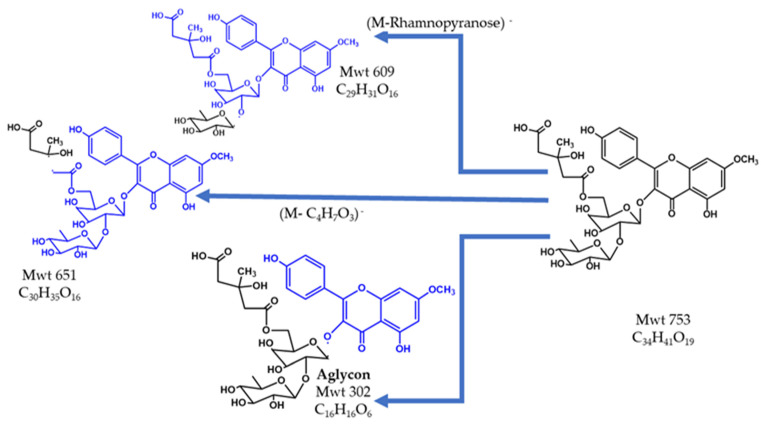
The structure and fragments of QueHMG.

The peak eluted at Rt 18.9 and *m*/*z* 753.3 using the ESI - Ve mode was the (M–H)^−^ (C_33_H_37_O_20_)^−^ of quercetin–3–*O*–α–l–rhamnopyranosyl–(1→2)–[6–*O*–(3–hydroxy–3– methylglutaryl)–β–d–galactopyranoside] (QueHMG), which was confirmed by the MS^2^ spectrum where the base peak at *m*/*z* 609.2 was due to the loss of the rhamnopyranosyl terminal sugar unit. The same peak at *m*/*z* 609.1462 was observed in the MS^2^ spectrum of QueHMG, identified in *A. monspessulanus* using high resolution ESI–MS (HRESIMS) [41]. Another peak in the MS^2^ spectrum at *m*/*z* 651.1(M–C_4_H_7_O_3_)^−^ was due to cleavage of the 3–hydroxy–3–methyl glutaryl moiety through McLafferty rearrangement at the carboxylic group attached to the sugar moiety. The genin was observed as a daughter peak at *m*/*z* 302 (M–rhamnopyranose– gluco–pyranose–H_2_O)^−^. Figure 5 shows the chemical structure and fragmentation of QueHMG.

**Figure 6:**
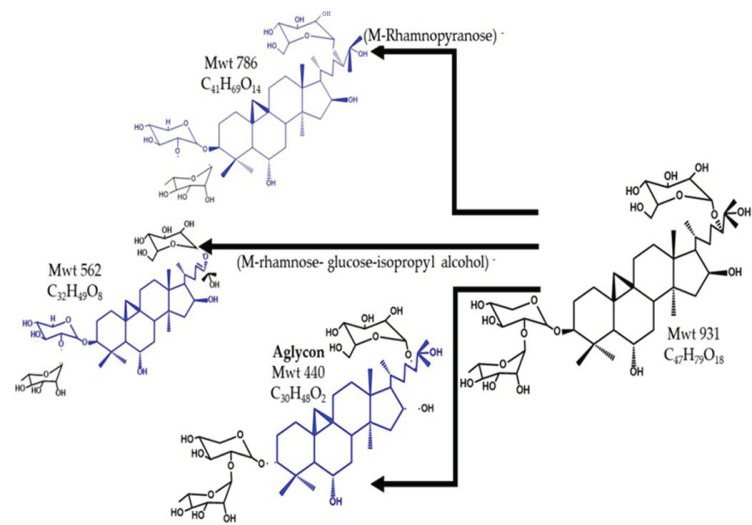
The structure and fragments of trojanoside C.

The component eluted at Rt 20.7 and at *m*/*z* 931 using the ESI - Ve mode was identified as the (M–H)^–^ of 3–*O*–[α–l–rhamnopyranosyl–(1→2)–β–d–xylopyranosyl]–24–*O*–β–d–glucopyranosyl–3β,6α,16β(24*S*),25–pentahydroxycycloartane, a cycloartane–type triglycoside saponin that was isolated and fully characterized from *A. trojanus*, and named as trojanoside C [42]. The MS^2^ spectrum showed a base peak at *m*/*z* 786 due to the loss of the terminal rhamnopyranosyl unit (M–146)^−^. The loss of isopropyl alcohol from the side chain created the daughter peak at *m*/*z* 869, and while that at *m*/*z* 562 was due to the loss of the terminal rhamnopyranose from C_3_, glucopyranose and isopropyl alcohol from C_24_ in the side chain (M–146–162–60)^−^. Figure 6 illustrates the chemical structure and fragmentation of trojanoside C.

**Figure 7:**
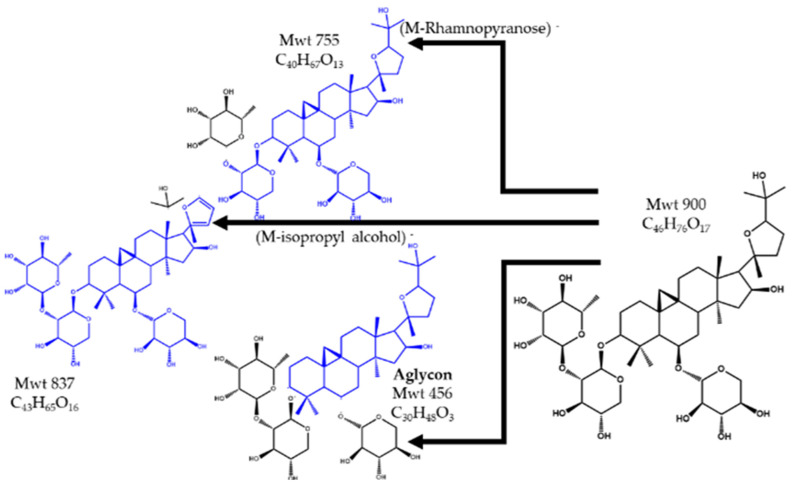
The structure and fragments of Astrasieversianin XV.

The peak at Rt 21.3 and *m*/*z* 900 obtained using the ESI - Ve mode was identified as (M–H)^−^ of 3–*O*–[α–l–Rhamnopyranosyl–(1→2)–β–d–xylopyranosyl]– 6–*O*–β–d– xylopyranosyl–20(*R*), 24(*S*)–epoxy–3β,6α,16β,25–tetrahydroxycycloartane, a cycloartane-type triglycoside saponin that was isolated and fully characterized from *A. sieversianus* [43] and *A. trojanus* [44], and named as Astrasieversianin XV. The MS^2^ spectrum demonstrated daughter peaks at *m*/*z* 755 and *m*/*z* 837 due to the loss of the terminal rhamnopyranosyl unit and isopropyl alcohol from the side chain, respectively. The peak at *m*/*z* 798 (M–C_6_H_14_O)^−^ was created after cleavage of the side chain, resulting in a stable carbocation on the C_20_ carboxylic carbon. Figure 7 shows the chemical structure and fragmentation of Astrasieversianin XV.

**Figure 8:**
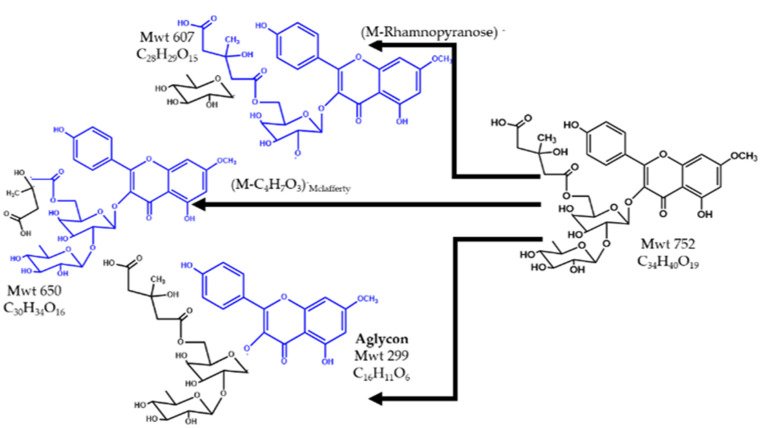
The structure and fragments of MethKaeHMG.

One of the main components eluted at Rt 22.5, *m*/*z* 751.5 using ESI - Ve mode was identified as (M–H)^−^ of 7–Methylkaempferol–3–*O*–α–l–rhamnopyranosyl–(1→2)–[6–*O*– (3–hydroxy–3–methylglutaryl)–β–d–galactopyranoside] (MethKaeHMG). The MS^2^ showed a base peak at *m*/*z* 607 due to the loss of the rhamnopyranosyl unit. The peak at *m*/*z* 689 might arise from the loss of one methoxy and two hydroxyl groups from the flavonoidal nucleus. Another fragment at *m*/*z* 650 (M–C_4_H_7_O_3_)^−^ emerged from cleavage of the 3–hydroxy–3–methyl glutaryl moiety through McLafferty rearrangement at the carboxylic group attached to the sugar moiety. The aglycon peak of 7–methoxy kaempferol was observed at m/z 299. The peak at *m*/*z* 607 in the MS^2^ spectrum of MethKaeHMG was previously observed while studying the flavonoidal constituents of *A. gombiformis* [40]. Figure 8 provides the chemical structure and fragmentation of MethKaeHMG.

#### 2.1.2. Compounds Tentatively Identified Using APCI Mode

**Figure 9:**
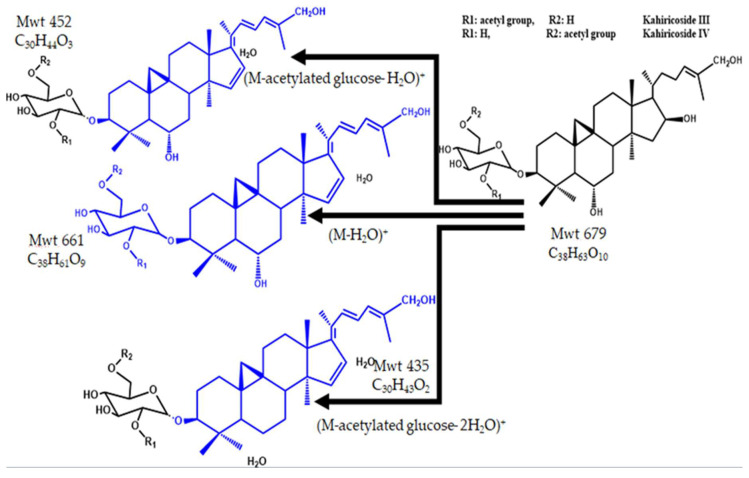
The structure and fragmentation of kahiricoside III and IV.

The two main components eluted at Rt 20.9 and 22.2 at *m*/*z* 679 were identified as (M+H)^+^ of kahiricoside III and IV, respectively. Both are cycloartane-type saponin glycosides attached to acetylated glucose, where the acetyl group is attached to C_2_ in kahiricoside III and C_6_ in kahiricoside IV. They were previously isolated and fully characterized from *A. kahiricus* where the (M+H)^+^ was determined by high resolution fast atom bombardment (HRFAB)–MS at *m*/*z* 679.4412 for C_38_H_62_O_10_. Kahiricoside III was eluted first, followed by kahiricoside IV using C_18_ reversed-phase HPLC and methanol–water elution [15]. The MS^2^ spectrum indicated daughter peaks at *m*/*z* 661 and *m*/*z* 452 due to the loss of water and the acetylated glucose moiety with one water molecule, respectively. Those at *m*/*z* 643 and *m*/*z* 435 were due to the loss of two water molecules and the acetylated glucose compartment and two water molecules, respectively. Figure 9 shows the chemical structure and fragmentation of kahiricoside III and IV.

**Figure 10:**
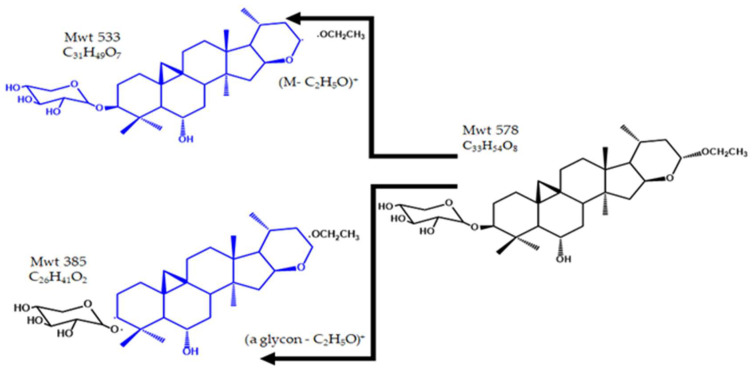
The structure and fragmentation of deacetyl tomentoside I.

The peak eluted at Rt 34.6 and *m*/*z* 579 using APCI + Ve mode was identified as (M+H)^+^ of deacetyl tomentoside I, which was previously isolated and fully characterized from the Egyptian *A. tomentosus*. The daughter peak at *m*/*z* 533 was observed earlier in the MS^2^ spectrum of deacetyl tomentoside I isolated from *A. tomentosus*, probably caused by loss of the ethoxy group C_2_H_5_O from the side chain. The peak at *m*/*z* 385 represents the genin compartment with the dissociation of the ethoxy group. Figure 10 shows the structure and fragmentation of deacetyl tomentoside I.

**Figure 11:**
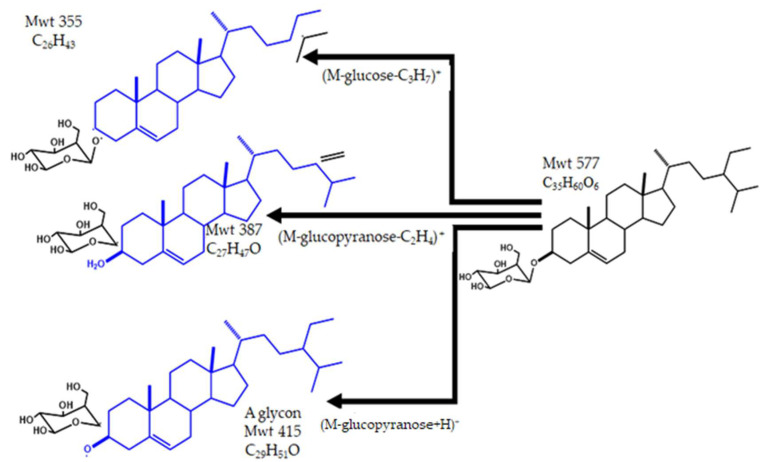
The structure and fragmentation of *β*–sitosterol–*β*–*d*–glucoside.

The peak at Rt 44.4 and *m*/*z* 577 using APCI + Ve mode was identified as the (M+H)^+^ of β–sitosterol–β–d–glucoside. In the MS^2^ spectrum, the peak at *m*/*z* 415 (genin) represents the cleavage of the glycosidic linkage and loss of the glucopyranosyl unit (M–162). Loss of C_2_H_4_ from the genin moiety’s side chain. The formation of a secondary carbocation in the side chain was also observed at *m*/*z* 387 (M–162–28)^+^. The loss of the sugar and C_3_H_7_ from the genin side chain probably resulted in the peak at *m*/*z* 355. β–sitosterol–β–d–glucoside was previously isolated from *A. tomentosus* [24], *A. tanae* [45]*, A. sieversianus* [47] and *A. altaicus* [46]. Figure 11 illustrates the structure and fragmentation of *β*–sitosterol–β–d–glucoside. 

### 2.2. Cytotoxic Activity

**Figure 12:**
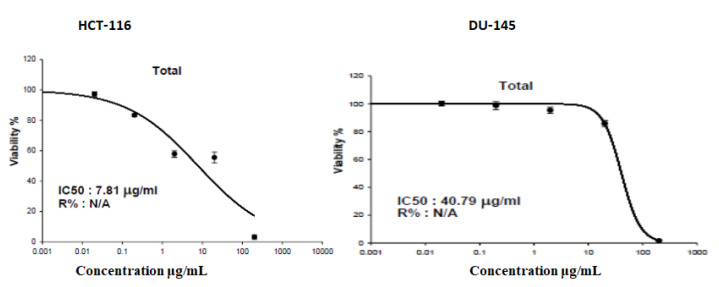
Dose response curves for the methanolic extract of *A. fruticosus* against HCT–116 and DU–145 cell lines.

Several natural products can prevent or even treat various cancers. Medicinal plants have been used to treat cancer as they can prevent or delay cancer onset, improve the immune system and the physiological status (35). Table 3 summarizes the cytotoxic screening of *A. fruticosus* leaf methanolic extract on seven cancer cell lines using concentrations ranging from 10 μg/mL–100 μg/mL. Following the preliminary cytotoxic screening, we generated dose response curves for different concentrations (0.02, 0.2, 2, 20 and 200 μg/mL) of the extract against colorectal (HCT–116) and prostate (DU–145) cancer cells, the most sensitive cell lines, (Figure 12) with IC_50_ values of 7.81 μg/mL and 40.79 μg/mL, respectively. Meanwhile, the results for different concentrations of the methanolic extract against HCT–116 and DU–145 cells are represented in Supplementary Tables S1 and S2. At 10 μg/mL, the leaf methanolic extract was not cytotoxic against all the tested cancer cell lines, with the cell viability ranging from 96% to 102%. However, at 100 μg/mL, this extract exhibited prominent cytotoxicity against the colorectal cancer cells (HCT–116) with only 3.368% cell viability. It also showed relatively moderate cytotoxicity against prostate (DU–145), ovarian (SKOV–3) and lung (A–549) cancer cell lines with cell viability of 14.25%, 16.02% and 27.24%, respectively. The breast cancer (MCF–7) cells were the most resistant (54.1% cell viability), while the osteosarcoma (MG–63) and hepatocellular cancer (HepG2) cells exhibited cell viability of 39.09% and 37.13%, respectively.

These results are consistent with previous studies on the *Astragalus* species in Egypt where the ethyl acetate fraction of the *A. sieberie* whole plant methanolic extract showed higher cytotoxicity against HCT–116 cells than MCF–7 cells with IC_50_ 32.2 and 69.6 µg/mL, respectively [13]. Moreover, compounds isolated from *A. spinosus* roots [48] and *A. kahiricus* aerial parts exhibited cytotoxicity against the ovarian cancer cell line (A2780) with IC_50_ ranging from 16–47 µg/mL [15]. The methanolic extract of *A. fruticosus* showed dose dependent cytotoxicity against the two most sensitive cell lines, HCT–116 (colorectal) and DU–145 (prostate) cancer cells. The extract showed moderate cytotoxic activity against DU–145 cells (IC_50_ = 40.79 µg/mL), while that against HCT–116 cell line was significant (IC_50_ = 7.81 µg/mL). Figure 12 represents the dose response curves for the methanolic extract of *A. fruticosus* against HCT–116 and DU–145 cell lines.

### 2.3. Antioxidant Activity

DPPH is a stable radical compound with an intense violet color that fades upon reaction with antioxidants [49]. We found 21.05% reduction in the absorbance of the DPPH radical, indicating weak to intermediate antioxidant capacity. The percentage scavenging of DPPH radical was then expressed as trolox equivalent antioxidant capacity (TEAC) that was calculated as approximately 2.51 μg/mL from the calibration curve. This shows that the percentage scavenging activity of 100 µg/mL of the extract was equivalent to the antioxidant potential of 2.31 μg/mL of trolox. Combination of natural products eliminates toxic side effects of chemotherapy and decreases colon cancer incidence [50]. This is consistent with studies showing low to intermediate antioxidant potential of some members of Astragalus growing in Egypt, such as *A. sieberi* [13] and *A. bombycinus* [18].

## 3. Materials and Methods

### 3.1. Plant Materials

The aerial parts of *Astragalus fruticosus* were collected during the flowering stage from Rashid, 40 km east of Alexandria, Egypt in March 2021 [31°23′07.7″ N, 30°25′13.5″ E]. The plant was kindly identified by Professor Sherif Sharawy, Professor of Plant Taxonomy, Faculty of Science, Ain Shams University. A voucher specimen (a.f.001) was deposited in the herbarium of the Pharmacognosy Department, Faculty of Pharmacy, Damanhour University. After air–drying, 10 gm of the aerial parts were pulverized and extracted with 100 mL absolute methanol using the Soxhlet apparatus at 70 °C. The methanolic extract was evaporated to dryness using a rotary evaporator at 60 °C.

### 3.2. HPLC–ESI/APCI–MS/MS Analysis

HPLC separation was performed using the Eclipse XDB C18 column (50 × 2.1 mm, 1.8 μm) (Agilent, Santa Clara, California, USA) using an HPLC system (Agilent HP 1100, USA). The mobile phase was composed of (A) methanol with 0.05% formic acid and (B) water with 0.05% formic acid. The compounds were separated using gradient elution profile: 0 min, A:B 10:90; 36 min, A:B 100:0; 50 min, A:B 100:0. Post-run time, 16 min. Chromatography was performed at 30 °C with a flow rate of 0.3 mL/min using the Bruker daltonik mass spectrometer (Bremen, Germany) equipped with ESI and APCI interfaces. For ESI, the ionization was on negative ion mode with Turbo Spray source, scan type Q1 MS 50–1200 *m*/*z*, scan rate 2000 Da/s, CUR gas 25, temperature 450 °C, gas 1 50, gas 2 40 and ion spray voltage 4500 V. For APCI, the ionization was on positive ion mode; the settings for the nitrogen drying and nebulizer gas were 5 mL/min (325 °C) and 60 psi. The APCI temperature and the capillary amperage were investigated before optimizing other parameters. Data analysis was performed using LC/MSD Trap Software 5.3 (Bruker daltonik). The compounds were tentatively identified by constructing an in-house mass spectra (MS/MS^2^) library of various *Astragalus* compounds from literature and comparing their molecular weights and fragmentation (MS/MS^2^).

### 3.3. Cytotoxicity Assay

We used 10 and 100 μg/mL of the leaf methanolic extract in DMSO to perform the preliminary cytotoxic assay against seven cancer cell lines. The ovarian (SKOV-3) and colorectal (HCT–116) cancer cells were maintained in RPMI media, while the others (lung A–549, prostate DU–145, breast MCF–7, osteosarcoma MG–63 and hepatocellular HepG2 cancer cell lines) were cultured in DMEM media. Both were supplemented with 100 mg/mL of streptomycin, 100 units/mL penicillin and 10% of heat-inactivated fetal bovine serum in humidified, 5% (*v*/*v*) CO_2_ atmosphere at 37 °C [50]. Cell viability was assessed using the sulforhodamine B (SRB) assay [51,52]. In 96-well plates, 100 μL cell suspensions (5 × 10^3^ cells) were incubated in complete media for 24 h. The cells were treated with 100 μL media containing various concentrations of plant extracts. After treating for 72 h, the cells were fixed by replacing the media with 150 μL of 10% TCA and incubated at 4 °C for 1 h. The TCA solution was removed, and the cells were washed five times with distilled water. Then, 70 μL SRB solution (0.4% *w*/*v*) was added and incubated at room temperature for ten min in the dark. The plates were washed thrice with 1% acetic acid and allowed to air-dry overnight. Then, 150 μL of TRIS (10 mM) was added to dissolve the protein-bound SRB stain, and the absorbance was measured in triplicates at 540 nm using a BMG LABTECH^®^– FLUOstar Omega microplate reader (Ortenberg, Germany). All the cell lines were obtained from Nawah Scientific Inc., (Mokatam, Cairo, Egypt). After the preliminary screening, five different concentrations (0.02, 0.2, 2, 20 and 200 μg/mL) of the extract were tested for their cytotoxic activity against colorectal (HCT–116) and prostate (DU–145) cancer cell lines, the most sensitive cell lines. Dose response curves were generated, and the cytotoxic activity of the plant extract was expressed as IC_50_.

### 3.4. Antioxidant Activity of the Methanolic Extract

The antioxidant capacity of 100 μg/mL *A. fruticosus* methanolic extract was determined using DPPH assay based on the redox potential of DDPH (Sigma–Aldrich, St. Louis, MO, USA). We prepared 1 mM DPPH solution (0.394 mg/mL) in methanol and then diluted it 1:10 to obtain a 100 μM solution (Abs at 515 nm = 0.5–0.6). Then, 500 μL each of the test sample and 100 μM DPPH solution were mixed in a cuvette. The negative control contained 500 μL each of methanol and DPPH solution. Both solutions were incubated in the dark at room temperature for 15 min, and absorbance was read at 515 nm using methanol as blank. The results were expressed as the percentage reduction in the radical absorbance [18].

[Abs max (negative control) − (Abs sample + DPPH)/Abs max] × 100

The antioxidant potential of the extract was compared to trolox as a reference antioxidant agent, and the results were expressed as TEAC. We constructed the calibration curve between inhibition percentage and trolox concentrations (12.5–0.3 μg/mL). We obtained a high correlation coefficient value of 0.9915, which indicated good linearity. We calculated the TEAC of the extract by substituting the inhibition percentage of the tested extract in the regression equation of the calibration curve.

### 3.5. Statistical Analysis

Data were expressed as the mean ± SD. The results were calculated using one way analysis of variance followed by Tukey multiple comparisons test. GraphPad Prism software (version 5) was used for all statistical analyzes and creating graphs.

## 4. Conclusions

To the best of our knowledge, this study is the first HPLC–MS/MS chemical profiling of *A. fruticosus* that demonstrates the predominance of flavonoidal and cycloartane-type saponin glycosides. These results agree with previous studies showing that the genus *Astragalus* is rich in these compounds. The observed cytotoxic activity of the total methanolic extract against colorectal cancer cells might facilitate further research efforts to isolate cytotoxic agent(s) for disease management. Further studies are underway to isolate, characterize and evaluate the potential efficacy of these cytotoxic phytochemicals against colorectal cancer cells. We have also initiated tissue culture experiments to enhance the production of potential compounds from the endangered plant *A. fruticosus*.

## Figures and Tables

**Table 1:** Compounds identified from *A. fruticosus* and their HPLC–MS/MS^2^ data using ESI-Ve ionization.

Cpd No.	Rt	MS^1^	MS^2^ Data	Compound Name
**1**	13.0	640 (M-H)-	519 (M-ring A-H)- 477 (M-glucopyranose-H)- 316 (a glycon) (M-2 x glucopyranose)-	5-hydroxy isomucronulatol-2/,5/-di-*O*-glucoside [39]
**2**	17.1	579 (M-H)-	447 (M-arabinopyranose)- 429 (M-arabinopyranose-H_2_O)- 284.8 (a glycon) (M-glucopyranose-arabinopyranose)-	2/,4/-trihydroxy-flavone-8-*C*-α-arabinopyranoside-7-*O*-β-glucopyranoside [18]
**3**	17.7	737.5 (M-H)-	675 (M- acetic acid)- 635 (M-C_4_H_7_O_3_)-Mclafferty 593 (M-rhamnopyranose)- 285 (a glycon) (Kaempferol-H)-	kaempferol-3-*O*-α-l-rhamnopyranosyl-(1→2)-[6-*O*-(3-hydroxy-3-methylglutaryl)-β-d-galactopyranoside] [40]
**4**	18.7	753.3 (M-H)-	651.1 (M-C_4_H_7_O_3_)-Mclafferty 609.2 (M-rhamnopyranose)-, 302.0 (a glycon) Quercetin-	Quercetin-3-*O*-α-l-rhamnopyranosyl-(1→2)-[6-*O*-(3-hydroxy-3-methylglutaryl)-β-d-galactopyranoside] [41]
**5**	20.7	931 (M-H)-	869 (M-isopropyl alcohol)- 786 (M-rhamnopyranose)-, 562 (M-rhamnose-glucose-isopropyl alcohol)- 440 (a glycon) (M-rhamnose-glucose-H_2_O)-	Trojanoside C [42]
**6**	21.3	900 (M-H)-	837 (M-isopropyl alcohol)- 797 (M-C_6_H_14_O)-Side chain 755 (M-rhamnopyranose)- 456 (a glycon) (M-Rhamnopyranose-2 xylose)-	Astrasieversianin XV [43,44]
**7**	22.5	751.5 (M-H)-	689 (M-OCH3-2OH)- 650 (M-C_4_H_7_O_3_)- 607 (M-rhamnopyranose)- 299.1 (a glycon) 7-methoxykaempferol-	7-Methylkaempferol-3-*O*-α-l-rhamnopyranosyl -(1→2)- [6-*O*- (3-hydroxy-3-methylglutaryl)-β-d-galactopyranoside] [40]

**Table 2:** Compounds identified in *A. fruticosus* and their HPLC–MS/MS^2^ data using APCI + Ve ionization.

Cpd No.	Rt	MS^1^	MS^2^ Data	Compound Name
**8**	20.9	679 (M+H)+	661 (M-H_2_O)+ 435 (M-acetylated glucose-2H_2_O)+	kahiricoside III [15]
**9**	22.2	679 (M+H)+	661 (M-H_2_O)+ 643 (M-2H_2_O)+ 453 (a glycon-H_2_O)+ (M-acetylated glucose-H_2_O)+ 435 (M-acetylated glucose-2H_2_O)+	kahiricoside IV [15]
**10**	34.6	579.3 (M+H)+	533 (M-C_2_H_5_O)+ 385 (M-xylose-C_2_H_5_O)+	deacetyl tomentoside I [22]
**11**	44.4	577.5 (M+H)+	415 (M-glucopyranose) + (a glycon) 387 (M-glucopyranose-C_2_H_4_)+ 355 (M-glucopyranose-C_3_H_8_O)+	β-sitosterol-β-d-glucoside [45,46,47]

**Table 3:** Summary for the preliminary cytotoxic screening of *A. fruticosus* leaf methanolic extract.

Cell Line	Cell Viability %	
	10 µg/mL	100 µg/mL
Colorectal cancer HCT-116	76.3179	3.68
Prostate cancer DU-145	95.093	14.253
Ovarian cancer SKOV-3	96.0288	16.0264
Lung cancer A-549	97.5202	27.2388
Hepatocellular carcinoma HepG2	99.9973	37.1369
Osteosarcoma MG-63	101.836	39.0968
Breast cancer MCF-7	99.0308	54.104

## Data Availability

Data is contained within the article and the supplementary materials.

## Supplementary Materials

The following supporting information can be downloaded at: https://www.mdpi.com/article/10.3390/ph15111406/s1: Table S1. Cytotoxic activity of different concentrations of the methanolic extract against colorectal cancer cell line HCT–116; Table S2. Cytotoxic activity of different concentrations of the methanolic extract against prostate cancer cell line DU–145; Figures S1–S11. The mass spectra for tentatively identified compounds using HPLC–MS/MS showing glycon daughter peaks are provided in the Supplementary file.

## Abbreviations

HPLC: High performance liquid chromatography, MS/MS^2^: Tandem mass spectroscopy, ESI: Electrospray ionization, APCI: Atmospheric pressure chemical ionization, Cpd No.: Compound number, *m*/*z*: Mass to charge ratio, Rt: Retention time, M^+^: Molecular ion, DMSO: Dimethyl sulfoxide, SRB: Sulphorhodamine B, TCA: Trichloroacetic acid, μg/mL: microgram per milliliter, TRIS: tris(hydroxymethyl)aminomethane, RPMI: Roswell Park Memorial Institute, DPPH: 1,1-diphenyl-2-picryl-hydrazyl radical, QueHMG: quercetin-3-*O*-α-L-rhamnopyranosyl-(1→2)-[6-*O*-(3-hydroxy-3-methylglutaryl)-β-d-galactopyranoside], MethKaeHMG: 7-Methylkaempferol-3-*O-*α-l-rhamnopyranosyl-(1→2)-[6-*O*-(3-hydroxy-3-methylglutaryl)-β-d-galactopyranoside], KaeHMG: kaempferol-3-*O*-α-l-rhamnopyranosyl-(1→2)-[6-*O*-(3-hydroxy-3-methylglutaryl)-β-d-galactopyranoside].
