# Supplementary material for: Insights into HPLC-MS/MS Analysis, Antioxidant and Cytotoxic Activity of Astragalus fruticosus against Different Types of Cancer Cell Lines
Source: Pharmaceuticals (Basel). 2022 Nov 14;15(11):1406. doi: 10.3390/ph15111406 (PMC9693330; doi:10.3390/ph15111406)

Insights into HPLC-MS/MS Analysis, Antioxidant and Cytotoxic Activity of *Astragalus fruticosus* against Different Types of Cancer Cell Lines

Mohamed Fayez Dekinash [1], Tarek M. Okda [2,*], Ehab Kotb Elmahallawy [3,*], Fathy Kandil El-Fiky [4], Gamal Abd El Hay Omran [2], Emil Svajdlenka [5], Naief Dahran [6], Manal F. El-Khadragy [7], Wafa A. Al-Megrin [7] and El Moataz Bellah Ali El Naggar [1]

Supplementary Tables

Supplementary Table S1. Cytotoxic activity of different concentrations of the methanolic extract against colorectal cancer cell line HCT-116.

Concentration (µg/ml)	Cell viability percentage of HCT-116 cells<br>Mean ± SD
200	1.67389 ± 0.479
20	55.4931 ± 3.530
2	77.8864 ± 2.214
0.2	83.3333 ± 0.867
0.02	97.1453 ± 1.501

Supplementary Table S2. Cytotoxic activity of different concentrations of the methanolic extract against prostate cancer cell line DU-145.

Concentration (µg/ml)	Cell viability percentage of DU-145cells<br>Mean ± SD
200	2.09592 ± 0.720
20	85.7544 ± 2.127
2	96.3997 ± 2.136
0.2	98.6422 ± 2.816
0.02	100.222 ± 1.665

Supplementary Figures

Supplementary Figures S1-S11. Mass spectra for tentatively identified compounds using HPLC-MS/MS showing a glycon daughter peaks.

Figure S1. Mass spectrum of 5-hydroxy isomucronulatol-2/,5/-di-*O*-glucoside.

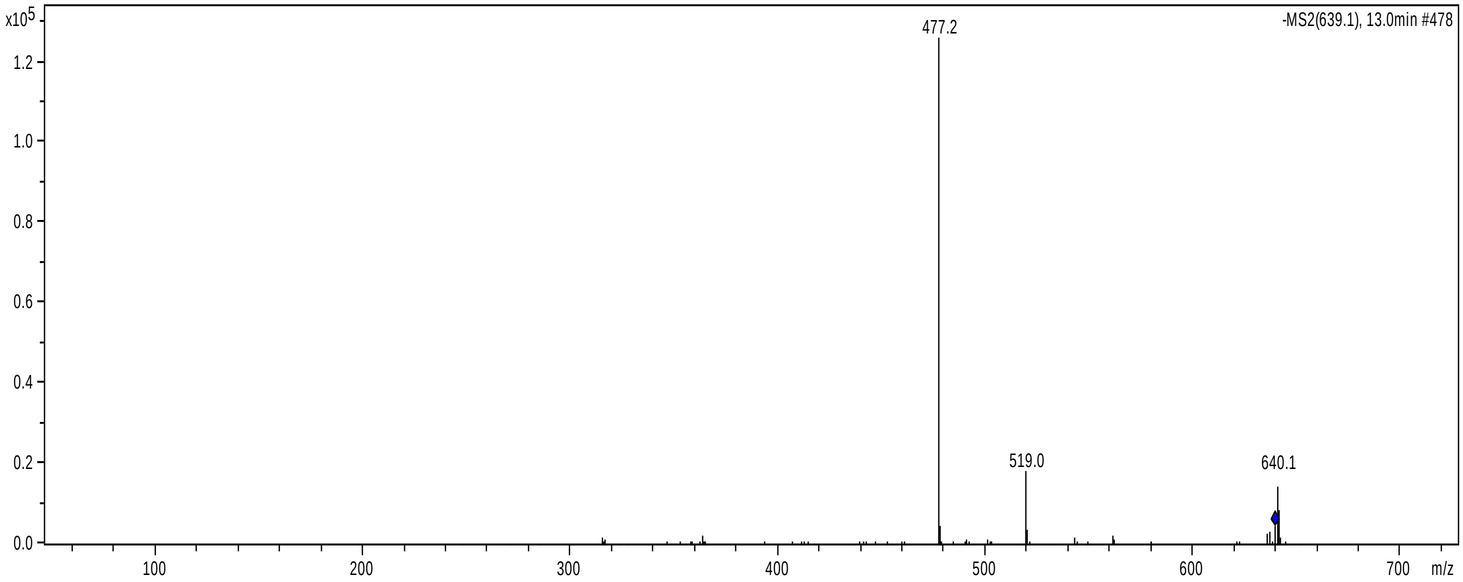

Figure S2. Mass spectrum of 2/,4/-trihydroxy-flavone-8-*C*-α-arabinopyranoside-7-*O*-β-glucopyranoside.

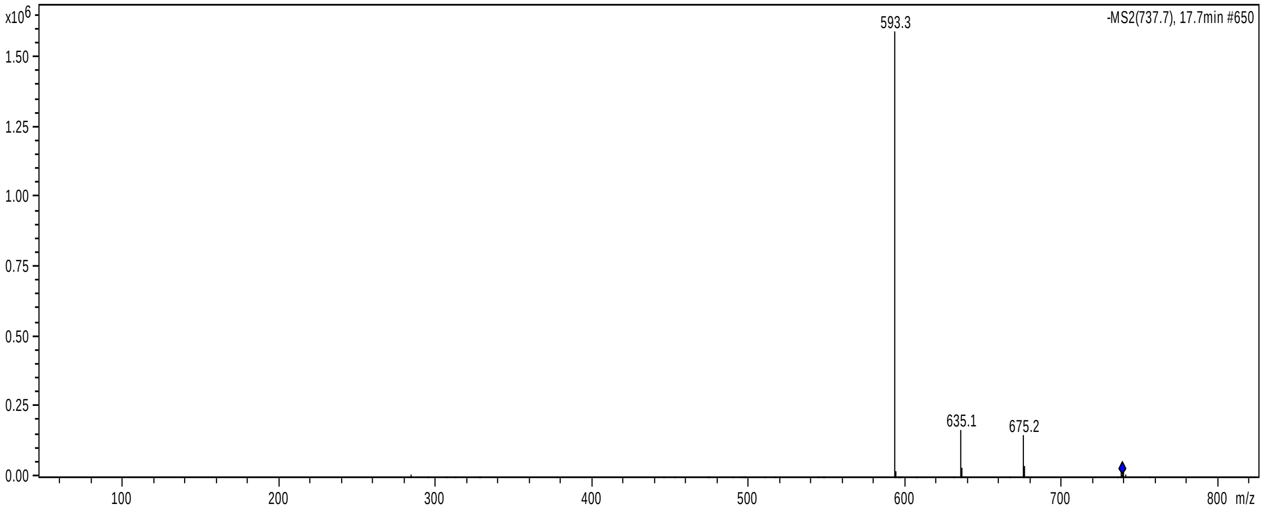

Figure S3. Mass spectrum of kaempferol-3-O-*α*-*L*-rhamnopyranosyl-(1→2)-[6-*O*-(3-hydroxy-3-methylglutaryl)-β-*D*-galactopyranoside].

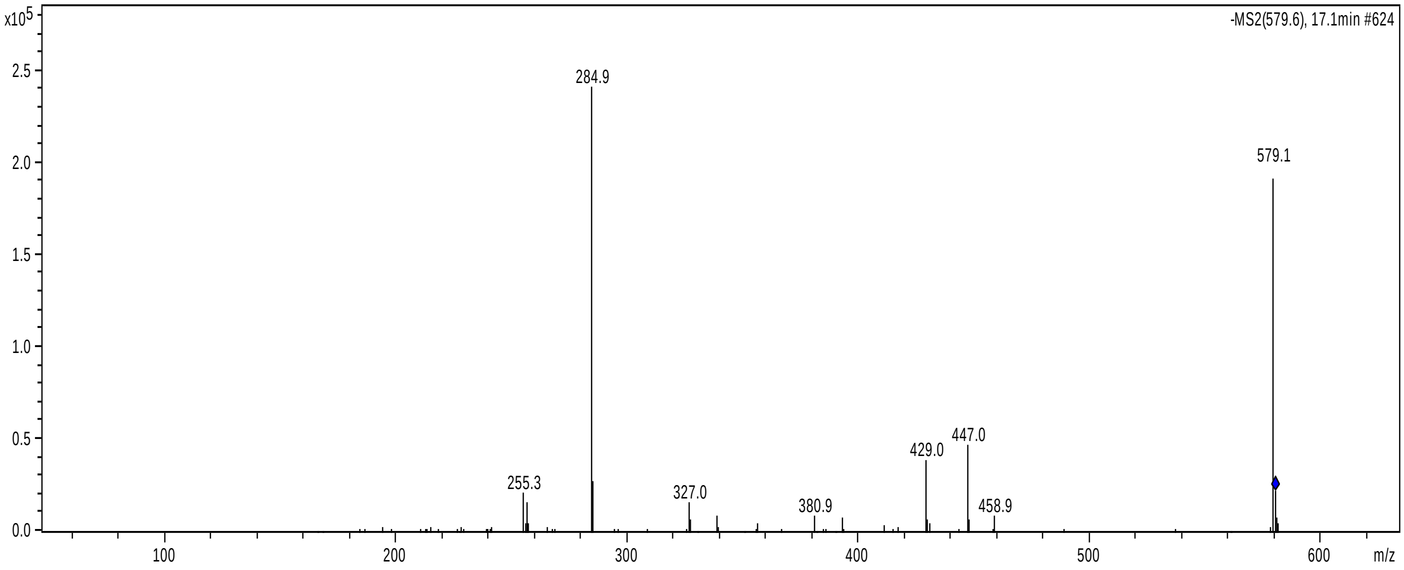

Figure S4. Mass spectrum of quercetin -3-O-α-L-rhamnopyranosyl-(1→2)-[6-O-(3-hydroxy-3-methylglutaryl)-β-D-galactopyranoside].

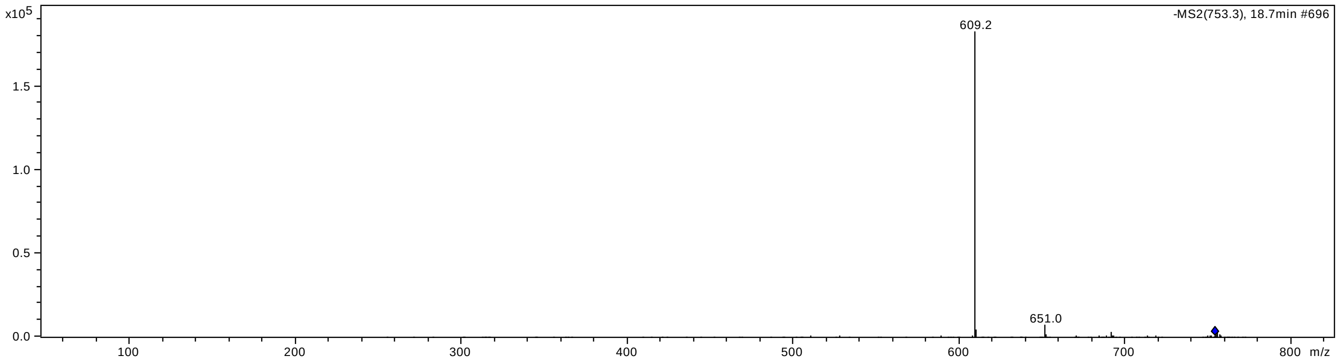

Figure S5. Mass spectrum of Trojanoside.

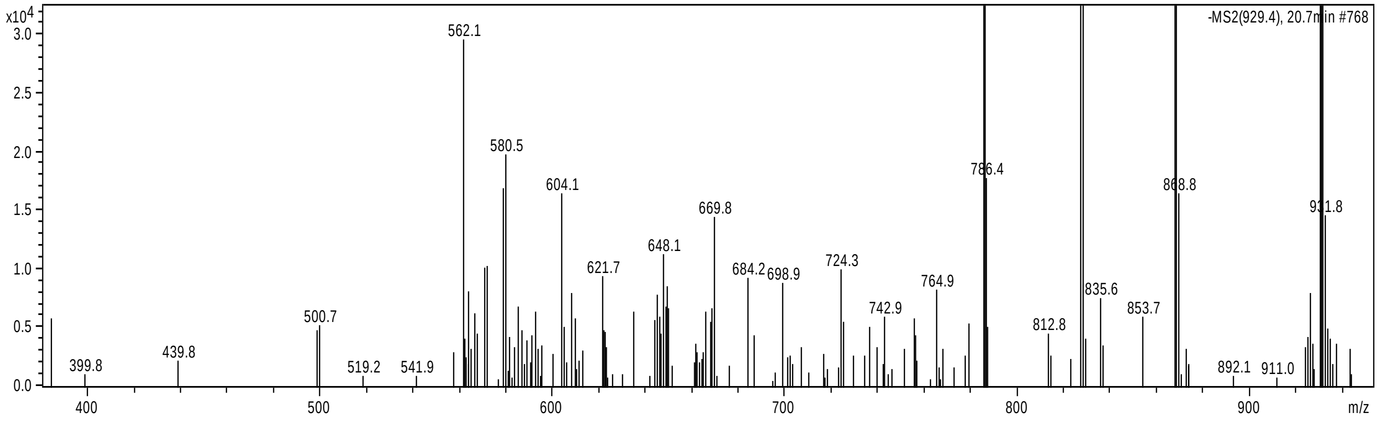

Figure S6. Mass spectrum of Astrasieversianin XV

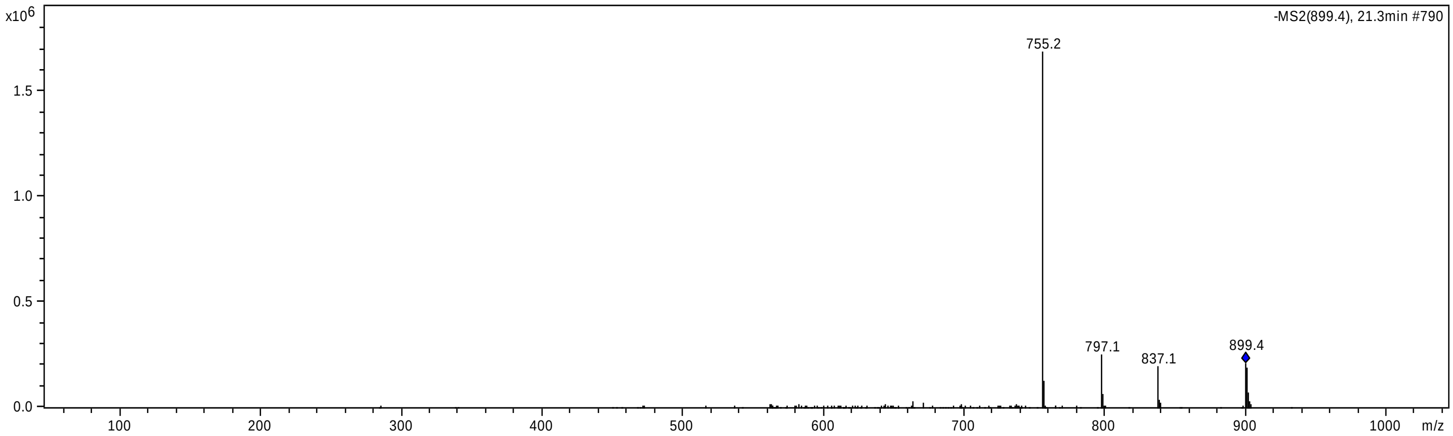

Figure S7. Mass spectrum of 7-Methylkaempferol-3-*O*-*α*-L-rhamnopyranosyl-(1→2)-[6-*O*-(3-hydroxy-3-methylglutaryl)-β-*D*-galactopyranoside].

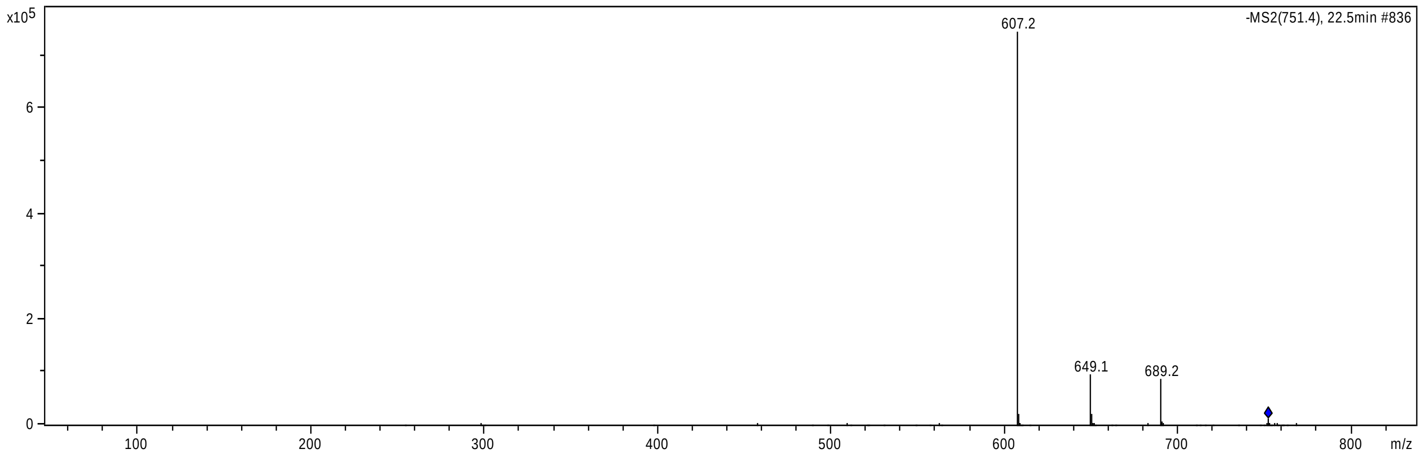

Figure S8. Mass spectrum of kahiricoside III.

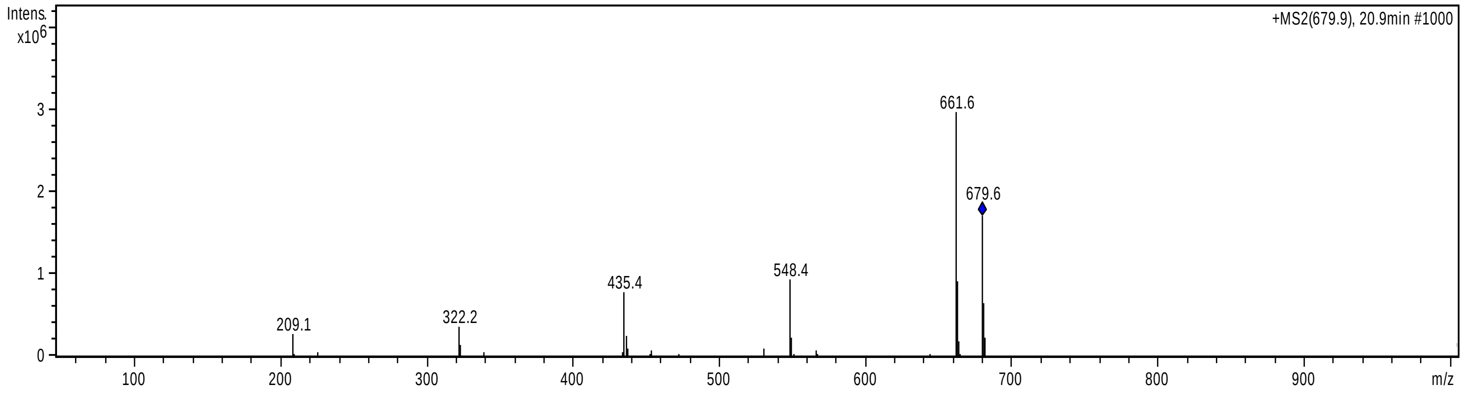

Figure S9. Mass spectrum of kahiricoside IV

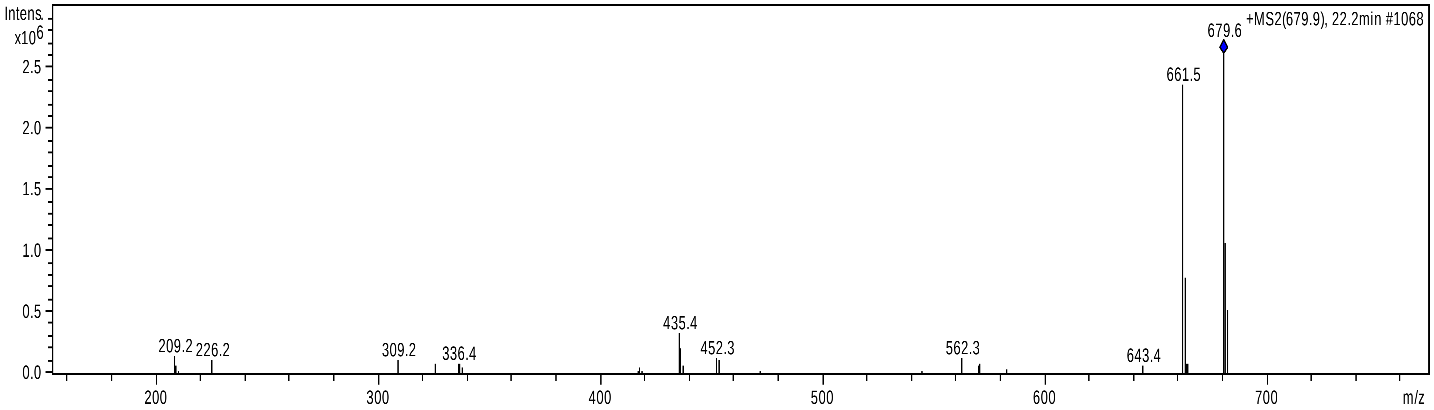

Figure S10. Mass spectrum of deacetyl tomentoside I

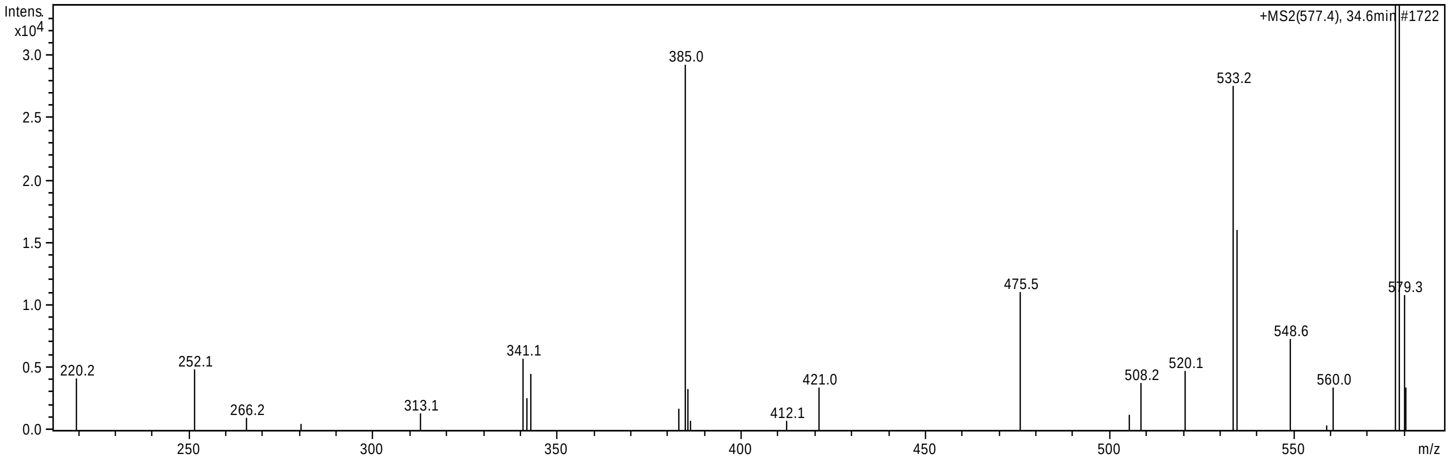

Figure S11. Mass spectrum of β-sitosterol-β-D-glucoside showing a glycon signal at m/z 385.

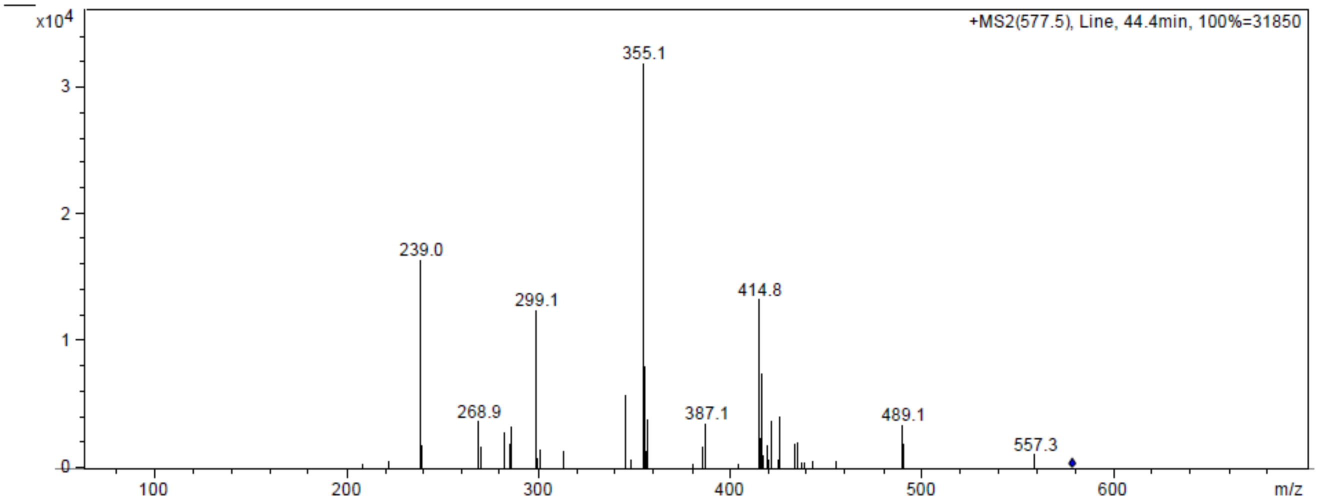

Supplement: Supplementary file 1 [file pharmaceuticals-15-01406-s001.zip › pharmaceuticals-1989737-supplementary.pdf]
